# Book review: Electrical fields and the experienced life

**DOI:** 10.3389/fncom.2024.1462913

**Published:** 2024-08-15

**Authors:** Christof Koch

**Affiliations:** ^1^Brain & Consciousness, Allen Institute, Seattle, WA, United States; ^2^Tiny Blue Dot Foundation, Santa Monica, CA, United States

**Keywords:** LFP (local field potential), cortex, EEG, current flow, extracellular potential

Consider what happens following a cardiac arrest. When the pulse of life ceases, nervous tissue stops receiving oxygen-carrying blood, causing the victim to lose consciousness within seconds. The brain's electrical activity, as assayed by electroencephalogram (EEG) electrodes, diminishes until the wiggling waves eventually turn isoelectric. At this point, the mind is extinguished—no more seeing, hearing, thinking, or hoping. Despite proliferating accounts of near-death experiences, there is no evidence that these often highly meaningful events take place in a brain, including its deeper structures, that has truly flatlined. Rather, it is far more likely that they occur afterward, as the brain boots up during and following the traumatic events accompanying resuscitation, resulting in mistimed experiences. It is the electrical activity of the nervous system and its constitutive neurons, glial, astrocytes, and other cellular actors that support the mind and behavior.

Humanity only discovered that animals and their brains run on electricity, rather than on fluids, in the late eighteenth century (by the Italian physician Luigi Galvani experimenting with twitching frog muscles). The action potential, a brief (< 1 ms) and small (< 0.1 V) excursion of the voltage across the neuronal membrane separating the inside of cells from the outside, was not recorded until the middle of the nineteenth century (the “nerve current” of Emil du Bois–Reymond). Action potentials, or “spikes” in the lingo, are the primary means by which information is relayed between nerves and from there to muscles. The underlying brief changes in the conductance of the membrane to specific ions were not analyzed until the first half of the twentieth century. This understanding found its first efflorescence in Alan Hodgkin and Andrew Huxley's elucidation of the biophysical mechanisms underlying the initiation and propagation of the action potential in the squid giant axon. They formulated a quantitative model that reproduced their observations in terms of time- and voltage-dependent membrane-bound conductances (one each for sodium and potassium ions, in parallel to a constant “leak” conductance). Hodgkin and Huxley took 3 weeks, on a hand-cranked calculator, to solve for the propagation speed of the action potential, which was within 10% of their observed value. This astounding feat earned them a Nobel Prize a decade later.

*Electric Brain Signals* demonstrates how far modeling of nervous tissue has matured over the ensuing seventy-plus years. Jointly composed, in a single melodious voice, by Gaute Einevoll, a well-known and genial physicist cum neuroscientist, and five of his former students at the Norwegian University of Life Sciences and the University of Oslo, the book allows the reader to develop physics-informed intuitions of how the microscopic currents from about 16 billion nerve cells of the human neocortex, the highly convoluted outermost portion of the brain, sing the body electric, the macroscopic electrical potential measured inside the head with implanted microelectrodes and outside the scalp with EEG electrodes.

Modeling these fields is based on two assumptions: First, neuronal activity is characterized by currents, flowing through Hodgkin–Huxley-like membrane conductances, and emanating from all points along the dendrites, cell bodies, and axons of nerve cells. Second, on the scale of tens of micrometers and larger, the extracellular space—filled with salty water squeezed into the tiny, twisting spaces outside of cells—behaves like a resistive cytoplasm. That is, membrane currents superimpose linearly, and the resultant electrical potential is determined by volume conduction according to Ohm's law. Endogenous magnetic effects are negligible (due to the low velocity of the charged ions and the tiny magnetic permeability of brain tissue, about the same as the surrounding air). Everything else is a consequence of volume conduction. Neither fancy quantum effects between neurons (leaving open the possibility of intra- and inter-molecular quantum effects) nor other exotic physics appear to be relevant (although what is not disallowed by physics may, of course, be exploited by natural selection). Of course, the devil is in the details. Whether current sinks and sources superimpose cooperatively or destructively, how they contribute to the various EEG bands (delta, alpha, and gamma), depend on highly orchestrated synaptic volleys that excite and inhibit a densely interwoven neural lace of staggering intricacy. Taming this complexity, in tissue made from 160 billion cells of 5,000 highly heterogenous types—a challenge upon which the Human Brain Project foundered—is going to take all human ingenuity and then some.

The book facilitates this process using a series of highly instructive toy models (and Jupyter notebooks to accompany them) to guide the reader in interpreting spatial-temporal EEG signals as reflecting underlying neural activity patterns filtered through the cortical gray matter, the highly conductive cerebral spinal fluid, the skull, and the scalp. Other topics covered are electrodes that are implanted in patients and rest on top of the cortex, but underneath the skull, and modern magnetoencephalography (MEG) that tracks the very weak magnetic fields produced by brain activity at a higher spatial resolution than EEG.

Why, in the age of machine learning, should anyone care about the messy physics of biological tissue? Indeed, does the brain even care about its own biophysics? Hemodynamic activity in the heart generates sounds that can be picked up by a cardiologist to diagnose a heart murmur or an irregular rhythm. However, there is no evidence that these weak auditory signals are of any relevance to the body. What about the electrical fields generated by the brain? Do they affect its processing outside of epileptic seizures? In the closing chapter, *Electric Brain Signals* alludes to such *ephaptic* interactions in which endogenous field might affect not so much the occurrence but the timing of action potentials. Ephaptic interactions may, indeed, already be at work in the recently discovered temporal interference stimulation technique.

But more importantly, and contrary to the spirit of the times that sees the brain as nothing but a sophisticated computational engine, the nervous system is a physical organ like any other, subject to mechanical, metabolic, thermodynamic, and other physical constraints. If we are to comprehend its function and dysfunction, whether those are neurological or psychiatric, we must study the substrate that supports the mind at its mechanistic level. That is why *Electric Brain Signals* is essential reading for anyone who cares about the brain. As the rational design of the mRNA vaccine during the pandemic so vividly demonstrated, therapeutic interventions to ameliorate lost capabilities, such as blindness or quadriplegia, let alone enhancements via futuristic brain-machine interfaces, require mastery of the physics of the most excitable piece of active matter in the known universe.

## Author's note

CK is a neuroscientist at the Allen Institute and at the Tiny Blue Dot Foundation, the former president of the Allen Institute for Brain Science, and a former professor at the California Institute of Technology. His latest book is *Then I am Myself the World*.

## Author contributions

CK: Writing – original draft, Writing – review & editing.

